# Cannabis-Based Products for the Treatment of Skin Inflammatory Diseases: A Timely Review

**DOI:** 10.3390/ph15020210

**Published:** 2022-02-09

**Authors:** Ana M. Martins, Ana L. Gomes, Inês Vilas Boas, Joana Marto, Helena M. Ribeiro

**Affiliations:** 1 Research Institute for Medicines (iMed.ULisboa), Universidade de Lisboa, 1649-003 Lisbon, Portugal; hribeiro@campus.ul.pt; 2 Faculdade de Farmácia, Universidade de Lisboa, 1649-003 Lisbon, Portugal; analucia199867@gmail.com (A.L.G.); inesvb97@gmail.com (I.V.B.)

**Keywords:** *Cannabis sativa*, cannabinoids, CBD, THC, dermatological inflammatory diseases

## Abstract

The use of natural products in dermatology is increasingly being pursued due to sustainability and ecological issues, and as a possible way to improve the therapeutic outcome of chronic skin diseases, relieving the burden for both patients and healthcare systems. The legalization of cannabis by a growing number of countries has opened the way for researching the use of cannabinoids in therapeutic topical formulations. Cannabinoids are a diverse class of pharmacologically active compounds produced by *Cannabis sativa* (phytocannabinoids) and similar molecules (endocannabinoids, synthetic cannabinoids). Humans possess an endocannabinoid system involved in the regulation of several physiological processes, which includes naturally-produced endocannabinoids, and proteins involved in their transport, synthesis and degradation. The modulation of the endocannabinoid system is a promising therapeutic target for multiple diseases, including vascular, mental and neurodegenerative disorders. However, due to the complex nature of this system and its crosstalk with other biological systems, the development of novel target drugs is an ongoing challenging task. The discovery of a skin endocannabinoid system and its role in maintaining skin homeostasis, alongside the anti-inflammatory actions of cannabinoids, has raised interest in their use for the treatment of skin inflammatory diseases, which is the focus of this review. Oral treatments are only effective at high doses, having considerable adverse effects; thus, research into plant-based or synthetic cannabinoids that can be incorporated into high-quality, safe topical products for the treatment of inflammatory skin conditions is timely. Previous studies revealed that such products are usually well tolerated and showed promising results for example in the treatment of atopic dermatitis, psoriasis, and contact dermatitis. However, further controlled human clinical trials are needed to fully unravel the potential of these compounds, and the possible side effects associated with their topical use.

## 1. Introduction

Nowadays, several industries are moving toward plant-based ingredients, reflecting growing concerns with environment and sustainability. Additionally, this search for new phyto-ingredients to be used in the context of the skin is an innovation factor, much needed in order to stand out in a very competitive market [1,2]. One example is the increasing research on products based on *Cannabis sativa* (*C. sativa*, cannabis), which have gained prominence in recent decades, mainly due to the legalization of cannabis in an increasing number of countries. This is reflected in higher rates of growth and diversification of several cannabis-based products, including skin products [3]. However, some confusion remains regarding the benefits of these products, mainly due to the uncertainty in their compositions.

*Cannabis sativa* L. is an annual, pollinated, usually flowering plant from the Cannabaceae family. Many different varieties of this plant have developed throughout the centuries due to breeding and selection. However, due to the lack of a universally acknowledged taxonomic rank on the various groups of plants belonging to the genus Cannabis, it is commonly accepted to reference all types as *C. sativa* L. [4,5]. These plants originated with the first agricultural societies in Asia and have been used over the course of history for a wide variety of purposes, such as for fibers, food, oil, medicine, textiles, and also in recreational and/or religious practices [6]. The first medical uses of cannabis date back to when the emperor Chen Nung, the “father” of Chinese agriculture, drafted the first Chinese pharmacopoeia, in which cannabis was recommended for fatigue, rheumatism and malaria, and its seeds, due to their richness in γ-linoleic acid, were recommended to treat eczema, psoriasis, and inflammatory diseases. However, and despite all the knowledge on numerous applications and beneficial therapeutic effects of cannabis that were collected, documented, and shared between cultures throughout history, cannabis was banned in the twentieth century on account of its psychoactive effects [6,7,8]. Nevertheless, by the end of last century, there was a surge in the interest in cannabis, which is currently one of the fastest-growing products in agricultural markets. The expressions “hemp” or “industrial hemp” and “marijuana” or “medicinal cannabis” are broad classifications that were adopted into Western culture to differentiate between two types of the plant, with different purposes determined by different compositions. “Hemp” or “industrial hemp” are terms used to classify varieties of cannabis that contain 0.3% or less trans-Δ9-tetrahydrocannabinol (THC), the main psychoactive compound in the plant, while “marijuana” or “medicinal cannabis” can contain up to 30% of THC and is considered a controlled substance [9]. These low levels of psychoactive components are what make pharmaceutical industries bet largely on hemp to obtain the non-psychoactive cannabinoid cannabidiol (CBD), which has shown a high therapeutic value in numerous diseases. Therefore, among cannabis products for skin care, CBD oil (with high therapeutic potential and without undesirable psychotropic effects and extracted from the leaves) and hemp seed oil (which contains practically no cannabinoids (CNBs) in its composition and is extracted from the seeds) stand out [10].

Although most biological actions of cannabis are related to CNBs, it is worth mentioning that other *C. sativa* compounds can also have medicinal properties. Terpenoids, which have been identified in the flower, leaves and trichomes of the plant, seem to be responsible for the fragrance, and some protective functions of the plant. There are over 200 terpenoids identified in *C. sativa*, the most common being limonene, myrcene, and α-pinene, which are highly volatile compounds [6]. These molecules are easily extracted from the plant material by steam distillation, resulting in a substance called the essential oil or the volatile oil of the plant, or through vaporization [11]. Terpenes, closely related to terpenoids, have been associated with several medicinal properties including antimicrobial, antioxidant, anticancer, antiarrhythmic, antiaggregating, anesthetic, anti-inflammatory, and antihistaminic [12]. Some recent studies have also reported the synergistic contributions of terpenoids to cannabis-mediated effects, which can enhance CNB activity, thus making this matter is worth further investigation [11].

This review focuses on the possible therapeutic potential of cannabis, mainly the pharmacologically active cannabinoid compounds, for the treatment of skin inflammatory diseases. First, it introduces *C. sativa*, CNBs and the endocannabinoid system (ECS) in general, and then it focuses on the cutaneous ECS and on studies on the effects of CNBs in several skin inflammatory diseases, such as psoriasis, atopic dermatitis (AD), and allergic contact dermatitis (ACD). Finally, the last section focuses on legislation on cannabis use in medicine and current approved therapeutics.

## 2. Cannabinoids

**Figure 1:**
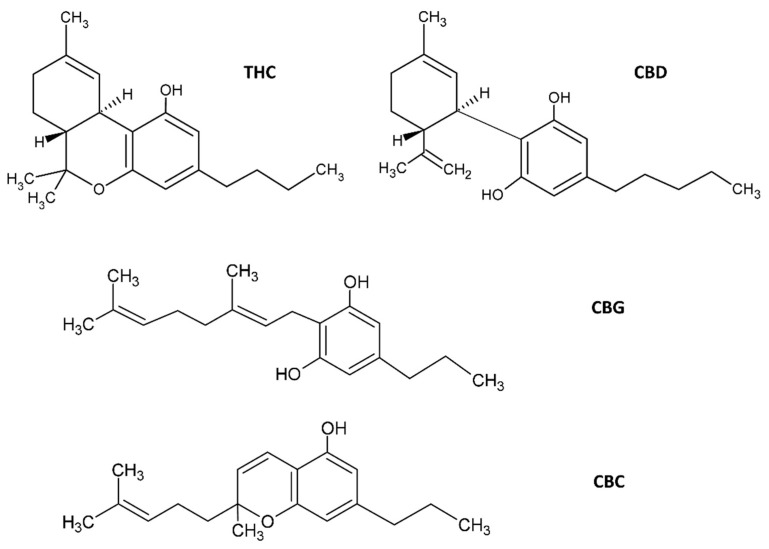
Molecular structures of *C. sativa* cannabinoids. The two main cannabinoids are CBD, cannabidiol and Δ9-tetrahydrocannabinol (THC). Additionally shown are CBG, cannabigerol and cannabichromene (CBC). Several other structures of phytocannabinoids are available in [14], for example.

Most therapeutic effects of *C. sativa* L. are due to the presence of CNBs, a broad term for a diverse array of compounds that have the common property of interacting with cannabinoid receptors (CBRs) [13]. This very heterogeneous group of pharmacologically active compounds are structurally and biochemically similar to the primary psychoactive compound derived from *C. sativa* L., THC. *C. sativa* contains approximately 565 different secondary metabolites [14], and approximately 120 are CNBs. Chemically, CNBs are terpenophenolic compounds, the best known being the previously mentioned THC and CBD [14,15,16]. Other pharmacologically important CNBs include cannabinoid acids, cannabigerol (CBG) and cannabivarins [17]. Cannabidiol and THC (Figure 1) are produced and deposited in the secretory and resinous cavity of the glandular trichomes of the plant, mainly found in pistillate flowers (female), in the bottom of the leaves and, occasionally, in the stems of young plants [14,16].

However, while THC has psychotropic effects and, therefore, has strict regulatory restrictions, CBD does not have such effects and has greater application freedom in the market [18]. Cannabidiol, a small 314 Da molecule, is one of the main pharmacologically active CNBs due to its antimicrobial [19], antioxidant and anti-inflammatory properties [20]. Although CBD is one of the most studied CNBs with therapeutic properties, the vast majority of phytocannabinoids (pCNBs) have little to no psychoactive activity, and most have acceptable side-effect profiles, which makes them particularly interesting candidates for the treatment of several diseases [21].

There are three main classes of CNBs, namely the pCNBs, exclusively produced by *C. sativa*, endocannabinoids (ECBs) which exist or are naturally produced in the human body, and synthetic CNBs which are similar to the pCNBs or ECBs but are lab synthesized [15]. Examples of each class are shown in Table 1.

Cannabinoids can also be distinguished based on whether they contain a carboxyl group, between neutral CNBs (such as THC and CBD) and cannabinoid acids. In plants, concentrations of neutral CNBs are much lower than those of cannabinoid acids, thus THC and CBD are formed by non-enzymatic decarboxylation, a consequence of stressful events, such as light exposure, heating, or ageing of their acidic precursors Δ9-tetrahydrocannabinolic acid (THCA) and cannabidiolic acid (CBDA) [22]. The amounts of each compound formed in the plant depend on genetic characteristics, and on environmental conditions, such as temperature, humidity, and soil nutrition. The enzymes THCA synthase and CBDA synthase were the first cannabinoid synthases to be studied, and are potential targets for several biotechnological applications, given that they produce the direct precursors of pharmacologically active CNBs [23,24].

## 3. The Endocannabinoid System

Endocannabinoids are, as previously mentioned, endogenous compounds that occur naturally, produced by humans and other animals, in the brain or peripheral tissues. They are typically referred to as neuromodulator agents and have characteristics that distinguish them from typical neurotransmitters: they are synthesized at will in their place of action, by receptor-stimulated cleavage of precursors of the lipid membrane and are not preserved in synaptic vesicles [15,25]. Endocannabinoids are arachidonic acid (AA) derivatives, the two best known being 2-arachidonoyl-glycerol (2-AG) and anandamide (AEA) [15,26,27]. Oleoylethanolamine (OEA) and palmitoylethanolamide (PEA) are members of the extended ECB family, and although sometimes not strictly considered ECBs because they do not bind the typical ECB receptors (CB1R/CB2R), they are known to bind other receptors (e.g., the nuclear peroxisome proliferator-activated receptors (PPARs)) and enhance the activity of AEA [28,29].

**Figure 2:**
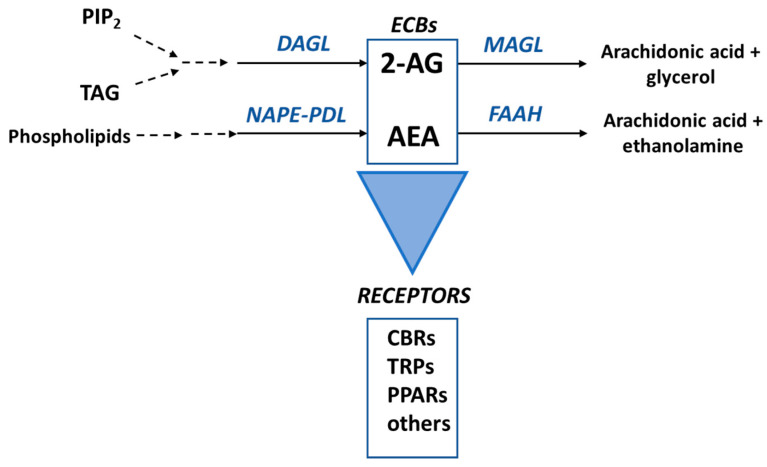
The main components of the endocannabinoid system. The principal enzymes involved in the biosynthesis of 2-arachidonoyl-glycerol (2-AG) and anandamide (AEA) are diacylglycerol lipase (DAGL) and N-acylphosphatidylethanolamine-phospholipase D (NAPE-PLD), respectively. These ECBs are mainly metabolized by monoacylglycerol lipase (MGL) and fatty acid amide hydrolase (FAAH), as shown. ECBs mainly bind to Cannabinoid Type 1 (CB1R) and Cannabinoid Type 2 (CB2R) receptors to exert their functions in the cells but can also bind to for example transient potential ion channels receptors (TRPs) and nuclear peroxisome proliferator-activated receptors (PPARs). PIP_2_, phosphatidylinositol-4,5-bisphosphate; DAG, diacylglycerol.

Endocannabinoids exert their functions via the endocannabinoid system (ECS), an evolutionarily conserved complex intercellular signaling network, which plays a role in the body homeostasis in humans [26]. This system, discovered in humans in the early 1990s, is composed of the signaling molecules (the endogenously produced ECBs), the proteins involved in their synthesis, catabolism and transport, and the ECB receptors [14,15,26,30,31] (Figure 2). The main functions of the ECS seem to be related to the modulation of the immune and nervous systems, and this system is involved in several physiological processes ranging from appetite and lipid metabolism to neurogenesis and neuroprotection [32].

The two main receptors for ECBs are the G-coupled proteins Cannabinoid Type 1 (CB1R) and Cannabinoid Type 2 (CB2R) receptors. However, ECBs can also bind to transient receptor potential (TRP) cation channels, peroxisome proliferator-activated receptors (PPARs) and ligand-activated ion channels for neurotransmitters such as serotonin (e.g., 5-HT1A, 5-HT2A and 5-HT3) [27]. In the ‘classical signaling pathway’, the binding of the CNB to CB1R/CB2R changes the receptors from an inactive to an active conformation, causing the dissociation of the Gα subunit of the G protein from the CBR and the Gβγ dimer. The Gα subunit then inhibits adenylate cyclase and the cAMP-dependent pathway. The dimer Gβγ, on the other hand, regulates mitogen-activated protein kinases (MAPKs) [32]. Endocannabinoids are lipid derivatives, thus they cannot be stored in vesicles and seem to be synthesized “on-demand” [33]. Phospholipids are the precursors of AEA, which is biosynthesized from these compounds via several different pathways, the main involving the enzyme N-acylphosphatidylethanolamine-phospholipase D (NAPE-PLD) [34]. This ECB is mainly metabolized by fatty acid amide hydrolase (FAAH), a membrane enzyme, to AA and ethanolamine [35]. The biosynthesis of 2-AG involves a signaling pathway starting with phosphatidylinositol-4,5-bisphosphate (PIP2), and a metabolic pathway from sn2-arachidonate-containing triglycerides. The main biosynthetic enzyme, converting diacylglycerol (DAG) into 2-AG, is diacylglycerol lipase (DAGL). Although there are several metabolic pathways for 2-AG, the predominant pathway consists of the hydrolysis of the ester bond into AA and glycerol, catalyzed by monoacylglycerol lipase (MAGL) (Figure 2) [36].

## 4. The Skin Endocannabinoid System and the Use of Cannabinoids as a Potential Treatment for Skin Inflammatory Diseases

### 4.1. The Skin Endocannabinoid System

The skin is the human body’s largest organ, and the first barrier between the external environment and the inside of the body, protecting it against pathogens and chemical, biological and/or radiation damage. In addition to its protective function, the skin also plays a major role in the immune, neurologic, and endocrine responses, being composed of an intricate multicellular communication network, in which the skin and its pilosebaceous units function as neuroimmunoendocrine organs, responding to external stimuli, neuropeptides and mediators released by neighboring cells. It is a complex and delicate process that is essential for maintaining skin homeostasis [30,37].

**Figure 3:**
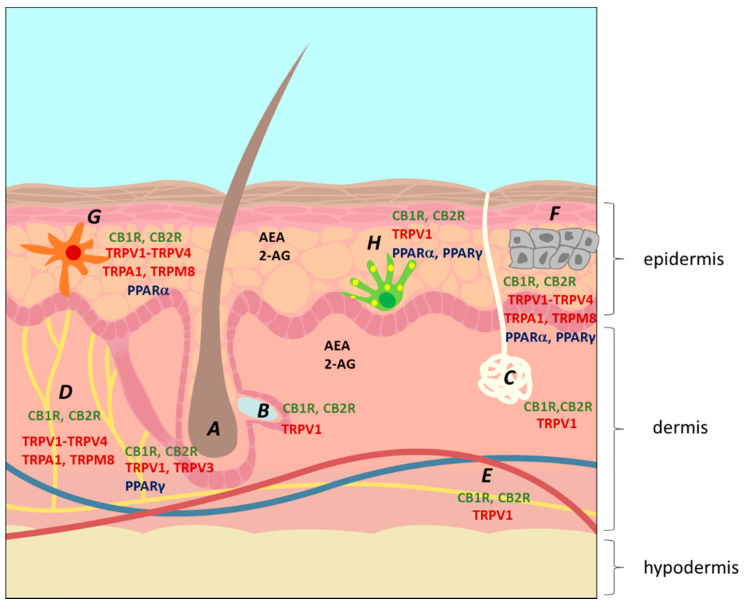
The location of the endocannabinoid system on the skin. A, hair follicle; B, sebaceous gland; C, sweat gland; D, nerve; E, blood vessels; F, keratinocytes; G, Langerhans cell (immunocyte); H, melanocytes. The figure shows the location of the two main ECBs, 2-arachidoynyl-glycerol (2-AG) and anandamide (AEA), and of several ECB receptors, including the main cannabinoid receptors CB1R and CB2R, several transient potential ion channel receptors (TRPs) and peroxisome proliferator-activated receptors (PPARs) [26].

Recently, it was suggested that the skin has its own ECS, since CB1R and CB2R were shown to have endogenous ligands in skin [38]. This ECS plays a critical role in the maintenance of skin homeostasis and barrier function, with ECBs being involved in the regulation of neuro-immunoendocrine skin functions [27]. This epidermal ECS possibly mediates the actions of ECBs in skin [39,40] and, when disrupted, may cause disorders such as dermatitis, acne, and pruritus [39,41,42,43,44,45]. The two main CNB receptors, CB1R and CB2R, have been found in epidermal keratinocytes, melanocytes, dermal cells, mast cells, sweat glands, hair follicles and cutaneous nerve fibers [26,46]. Other receptors have also been identified in several skin cells (Figure 3 and Table 2). Additionally, the enzymes FAAH and MAGL have been identified in sebocytes, melanocytes, fibroblasts and immunocytes, suggesting that skin is also involved in CNB metabolism [13].

Thus, an adequate pharmacological modulation of the cutaneous ECS by non-psychoactive CNBs, such as CBD, seems to be a feasible tool in the treatment of skin diseases, as will be further discussed.

### 4.2. Therapeutic Potential of C. sativa *L.* in Dermatology

Numerous studies have described how *C. sativa* L. can be used for chronic pain, spasticity, anorexia, nausea, and a plethora of other conditions and symptoms, including dermatological disorders, such as pruritus, and inflammatory skin diseases [49].

The discovery of a skin ECS led to the investigation of its role in the functions of this organ, and how disturbances in its regular actions can contribute to the development of pathological skin disorders [50]. Studies with CNB receptors, selective agonists, antagonists, and other regulatory agents that can regulate the levels and actions of ECBs during inflammatory processes have provided extensive evidence on the numerous immunomodulatory and anti-inflammatory effects of the ECS [38]. It also led to the suggestion that the use of these agonists, antagonists and regulators holds great potential as a possible treatment for several diseases of the skin.

It should be noted, however, that the benefits of cannabis in dermatology may also be due to other compounds, not only pCNBs. For example, hemp seed oil is a great skin protector, reducing dryness and slowing skin’s natural aging process, due to its high percentage of polyunsaturated fatty acids (PUFAs) [51,52], and significant amounts of vitamins and minerals, such as vitamin E [53]. This article, however, focuses on the actions of CNBs.

Cannabinoids are compounds of interest in dermatology due to their anti-inflammatory, antipruritic and antinociceptive properties [54]. The biological activity of CNBs in skin is a relatively recent area of research [27], even if cannabis preparations for topical use have been described in ancient medical literature, mainly due to their antibacterial activities [42]. Several studies have reported the action of these compounds in the treatment of acne vulgaris, allergic contact dermatitis, eczema, pruritus, psoriasis, skin cancer, etc. (reviewed in [15,26,27,41,55]). The most promising role for CNBs in dermatology seems to be the treatment of itch, either chronic itch or itch as a symptom of other diseases, such as AD, ACD, and psoriasis [49]. Endocannabinoids such as PEA and OEA have been shown to decrease pruritus, possibly by decreasing xerosis [56,57]. As discussed previously, PEA and OEA do not directly bind to CBRs but stimulate the activation of CB1R by AEA [28,29]. The anti-inflammatory properties of CNBs are also extremely useful for the treatment of skin inflammatory diseases. CNBs can modulate cytokine production and T-cell responses and, additionally, cell proliferation [44,58], making them promising therapeutic agents for example for psoriasis and acne.

The topical application of CNBs has the advantage of avoiding first-pass metabolism [59]. However, the possible use of CNBs in topical formulations is challenging since they are highly lipophilic compounds, thus poorly soluble in water [60], with a limited diffusion through skin [61], and are unstable, being susceptible to degradation by temperature, light and autoxidation [62]. These issues make CNBs good candidates for incorporation in advanced drug delivery systems (DDS), such as liposomes, nanoparticles, and micelles, to be applied topically or by other routes [17].

### 4.3. Research on the Use of Cannabinoids for the Treatment of Skin Inflammatory Diseases

This section reviews studies on the effects of CNBs and the modulation of the ECS in several skin inflammatory diseases. Table 3 summarizes such studies.

#### 4.3.1. Acne and Seborrhea

Acne and seborrhea are the most frequent dermatological disorders, both characterized by highly elevated sebum (lipid) production by the sebaceous glands (SGs). These glands play a central role in the regulation of cutaneous lipid homeostasis and in the development of the physical and chemical barrier. A dysregulation of the SGs may cause hypersecretion of sebum, leading to hyperproliferation of keratinocytes and sebocytes, and to the concomitant colonization by bacteria, mainly Cutibacterium acnes, in the obstructed pilosebaceous unit, causing acne vulgaris. C. acnes has been shown to trigger inflammatory reactions in the skin by inducing the expression of pro-inflammatory cytokines [64,89].

Cannabidiol has been suggested as a promising therapeutic agent for the treatment of acne vulgaris since it normalizes the lipogenesis of sebocyte cells (lipostatic effect, without compromising cell viability), decreases the proliferation of these cells (antiproliferative effect, without inducing sebocyte apoptosis) and decreases the levels of pro-inflammatory cytokines (anti-inflammatory effect) [64]. It is interesting that CBD has an opposite effect to that of ECBs. While ECBs stimulate the lipid synthesis in SGs via the ‘classical’ signaling pathway involving CB2R, CBD exerts a sebostatic (lipostatic and antiproliferative) action by activating TRPV4 receptors [42,64].

Dobrosi et al. [63] performed in vitro studies using cultured human SZ95 sebocytes and observed the presence of AEA and 2-AG in the cultures, and that the cells expressed CB2R but not CB1R. The ECBs increased lipid synthesis in a dose-dependent manner by upregulating genes involved in this process. Additionally, 2-AG and AEA induced apoptosis-driven cell death. These actions were mediated by selective CB2R-coupled signaling using the MAPK pathway. The authors suggested that agents that suppress the local output of these ECBs in the ailing SGs (e.g., DAGL inhibitors), and/or that inhibit CB2R on the sebocytes (CB2R antagonists), have therapeutic potential in the management of acne and seborrhea.

Additional in vitro studies using human sebocytes and human skin organ cultures (hSOC) provided evidence that CBD inhibited the lipogenic actions of several compounds (e.g., AA, combination of linoleic acid and testosterone), and suppressed the proliferation of human sebocytes [64]. The authors used hSOC to mimic the SG function in vivo and showed that CBD completely inhibited the lipogenic action of AEA in these experimental conditions. In pharmacological terms, CBD inhibited the AEA-induced prolipogenic ERK1/2 MAPK pathway by activating the transient receptor potential vanilloid-4 (TRPV4) ion channel. Gene expression studies showed that this led to the downregulation of genes related to lipid synthesis (NRIP1), which affects glucose and lipid metabolism, thus inhibiting sebocyte lipogenesis. Furthermore, it was observed that CBD had anti-inflammatory effects that seem to occur via upregulation of tribbles homolog 3 (TRIB3) and inhibition of NF-κB signaling, both dependent on the A2a adenosine receptor. The combined lipostatic, antiproliferative (TRPV4-dependent) and anti-inflammatory (A2a adenosine receptor-dependent) actions suggested CBD as a possible therapeutic agent for acne. In 2016, the same group [66] further described that, not only CBD, but other pCBs (CBC, CBDV, CBG, CBGV, and THCV) induced sebocyte apoptosis in vitro, at high concentrations (≥50 µM) and that, additionally, THCV inhibited their proliferation. Concerning basal lipogenesis, the results showed inhibition by CBC and THCV, while CBG and CBGV increased it, thus being pro-acne. Additionally, CBC, CBDV and THCV reduced AA-induced ‘acne-like’ lipogenesis. All tested pCNBs had anti-inflammatory properties, thus they were proposed as holding potential to manage skin inflammatory diseases.

An in vitro study by Jin and Lee [67] tested the antimicrobial, anti-inflammatory, and anti-lipogenic effects of hemp seed hexane extracts in human HaCaT keratinocytes. The extract showed antimicrobial activity against C. acnes and anti-inflammatory effects in C. acnes-stimulated HaCaT cells, by reducing the expression of genes encoding inflammatory enzymes (iNOS and COX-2) and inflammatory cytokines (IL-1β and IL-8) and regulating NF-κB and MAPK signal pathways. Additionally, the extracts inhibited 5-lipoxigenase and MMP-9 activity, thus promoting collagen biosynthesis in vitro. Furthermore, the extracts had anti-inflammatory and anti-lipogenesis effects in IGF-1-stimulated lipogenesis. The authors suggest that these hemp seed extracts can be used to treat acne vulgaris; however, they point out that the observed effects may be due to the high content of PUFAs (e.g., linoleic acid, oleic acid, and palmitoleic acid) in the extracts.

The transdermal penetration of CNBs has also been reported and confirmed, which elicits the possibility for these agents to be efficiently applied to the skin in topical pharmaceutical preparations, such as creams [50], facilitating the treatment of acne and other dermatological conditions. For example, Ali et al. [65] reported, in a single-blinded comparative study spanning over 12 weeks, that sebum levels and erythema significantly decreased with the use of a 3% cannabis seed extract cream on the right cheek twice per day, when compared to a control cream that was similarly applied on the left cheek. No irritant or allergic reactions were observed, thus the cream was considered safe. A phase 2 clinical trial was reported in the ClinicalTrials.gov, (accessed on 11 December 2021) website, where the effect of a topical solution containing up to 5% of CBD (named BTX 1503) was evaluated and compared to placebo in over 360 acne patients [68]. It was observed that there was a 40% acne reduction after 12 weeks of treatment with the CBD solution. All tested doses of CBD were safe, with no observed adverse effects.

In conclusion, targeting the skin ECS can help regulate sebum production and have therapeutical effects in both acne and seborrhea.

#### 4.3.2. Allergic Contact Dermatitis

Allergic contact dermatitis (ACD) is one of the leading causes of occupational diseases. This disease is caused by a type IV delayed hypersensitivity reaction that develops as an inflammatory response of skin exposed to specific allergens. This exposure induces a specific immune response, predominantly involving T cells and inflammatory cytokines, such as interleukin (IL)-6, IL-8 and tumor necrosis factor-alpha (TNF-α) [90,91]. In addition to avoiding triggers of the disease, the preferred treatment for ACD is the use of topical corticosteroids and calcineurin inhibitors, and systemic immunosuppressive agents for severe cases [91].

Concerning the therapeutic value of the use of CNBs in the treatment of ACD, studies with humans are still lacking, but mice studies revealed the involvement of CBRs, especially CB2R, in the inflammatory response of ACD, and proposed possible therapies involving this and other targets.

The studies of Ueda and co-workers [69] and, later, Oka et al. [70] and Karsak et al. [44] provided the first evidence of the anti-inflammatory effects of CNBs in mice models of ACD. Ueda et al. [69] subjected mice ears to a topical treatment with an ether-linked analogue of 2-AG and observed early ear swelling (0–24 h after challenge). A similar response but obtained later (1–8 days post-challenge) was observed when the ears were treated with a CBR2 agonist (HU-308). These responses were significantly decreased upon oral administration of CB2R antagonist (JTE-907) or inverse agonist (SR 144,528). Both JTE-907 and SR 144,528 significantly inhibited swelling caused by the contact allergen 2,4-dinitrofluorbenzene (DNFB). The results strongly support the involvement of CB2R in the local inflammatory response in ACD and suggest that CB2R antagonists/inverse agonists may be potential therapeutical agents against ACD. The study by Oka et al. [70] reported an increase in the concentration of 2-AG in a mice ear model of oxazalone-induced ACD. Treatment with a CB2R antagonist (SR 144,528), but not CB1R antagonist, suppressed the inflammatory response, emphasizing the role of CB2R in the process. Shortly after, a study by Karsak et al. [44] reported the involvement of another ECB, AEA, and both CB1R and CB2R in the response to ACD in a mice model. First, it was observed that mice with a double-knockout for both CBRs frequently scratched their ears. Additionally, the same knocked out animals had exacerbated allergic inflammation when treated with the contact allergen DNFB. Furthermore, wild-type animals treated with CBR antagonists also showed exacerbated inflammatory responses, which decreased in the presence of receptor agonists. In wild-type (WT) mice, it was observed that the subcutaneous or topical application of THC attenuated DNFB-induced ACD. Mice knocked out for the AEA catabolic enzyme FAAH had increased levels of AEA and reduced inflammation. Taken together, the results imply a role for the skin ECS in ACD, and the authors suggested that CBR agonists and FAAH inhibitors should be studied as potential therapies for this disease.

Another ECB, PEA, was also shown to be involved in ACD. This ECB enhances the activation of CBRs and TRPV1 receptors by AEA, directly activating the PPAR-α. Petrosino et al. [71] used a mice model (WT and double CB1R/CB2R knockout mutants) of DNFB-induced ACD to study the involvement of PEA in the inflammatory process. The results showed an increase in PEA levels and upregulation of TRPV1, PPAR-α and a PEA biosynthetic enzyme in ear keratinocytes. An intraperitoneal injection of PEA inhibited the DFNB-induced ACD inflammation in vivo, and this inhibition was reduced by TRPV1 antagonists. In vitro studies with HaCaT keratinocytes induced with polyinosinic polycytidylic acid (poly-(I:C)), which leads to the production of the monocyte chemotactic protein-2 (MCP-2) chemokine, showed increased levels of PEA and AEA. The MCP-2 chemokine is a pro-inflammatory mediator involved in the recruitment of macrophages and mast cells to inflammatory sites. Treatment with exogenous PEA inhibited the poly-(I:C)-induced expression and secretion of MCP-2, but this was reversed by TRPV1 antagonists, while PPAR-α and CB2R antagonists did not have any effect. In summary, the results suggest that endogenous production and exogenous administration of PEA may be protective against ACD development, and thus should be considered a possible therapy for this skin disease. A later study by the same group [73] provided the first evidence of the anti-inflammatory properties of CBD in an in vitro model of ACD, i.e., poly-(I:C)-stimulated human HaCaT keratinocytes. The results showed that treatment with CBD increased the endogenous levels of AEA and inhibited the production of the MCP-2 chemokine, IL-6, IL-8 and TNF-α more efficiently than treatment with other non-psychotropic pCNBs. This CBD anti-inflammatory effect could be reversed by a CB2R antagonist. Moreover, this effect was also antagonized by a selective TRPV1 antagonist, suggesting that CBD can also directly activate and desensitize this channel receptor. Because of the proven non-toxicity of CBD in humans, the authors suggested that this pCNB should be further tested in preclinical trials.

The anti-inflammatory effect of THC in DNFB-induced ACD was also reported but, in this case, it was independent of CB1R/CB2R [72]. The in vivo study used WT and CB1R/CB2R knocked out mice and showed decreased ear swelling after topical application of 12.30 µg of THC. The histological analysis revealed that THC decreased myeloid immune cell infiltration in both WT and mutant mice by inhibiting the secretion of IFN-γ by T cells, although not impairing the recruitment of these cells to the site of allergen challenge. In vitro studies using mice primary epidermal keratinocytes further revealed that THC inhibited the IFN-γ-induced production of chemokines by keratinocytes, and that this inhibitory action was responsible by the limited recruitment of myeloid cells.

Taken together, these studies suggest a possible role for cannabis in the treatment of ACD but further research is needed, especially trials with humans, since some contradictory results have been reported. For example, there was a report on the induction of ACD by cannabis in a woman who used it to treat chronic back pain [92].

#### 4.3.3. Asteatotic Eczema

Asteatotic eczema (AE), also known as eczema craquelé or xerosis (dry skin), is a common type of pruritic dermatitis. It is characterized by dry, scaly, cracked, and itchy skin, which is typically inflamed [74,93]. It usually begins as dry skin, and as the disease becomes more severe, the skin can crack and cause fissures, which led to epidermal water loss. The condition is often exacerbated by dry and cold weather, being associated with skin exposure to environmental irritants. As such, and because prevention is key in avoiding or controlling itch and irritation, patients are advised on several lifestyle alterations, such as avoiding harsh cleansing agents, and opting for lukewarm water showers, to prevent exacerbation of this uncomfortable disease [93]. Treatment for AE usually involves application of emollients containing urea, lactic acid or its salts. Severe cases, however, typically require topical corticosteroid treatment [74].

Symptoms seen in eczema and other forms of xerotic dermatitis are partially due to impaired skin barrier repair. Endocannabinoids such as PEA and AEA exist in high concentrations in the stratum granulosum of the skin, and low levels of these compounds have been linked to xerosis [94]. Several studies have provided evidence that the modulation of the skin ECS can lead to increased lipid synthesis in that skin layer, providing relief for eczematous conditions [50,74]. A clinical study by Yuan et al. [74], for example, reported that AE patients who received a 0.3% PEA/0.21% AEA cream showed significant improvement in itching and skin hydration, as well as a decrease in erythema, scaling, and dryness, typical of eczema and other skin diseases. The proposed mechanism is that ECBs enhance lipid production in the stratum granulosum [15]. No adverse effects were observed in any subject during the 28 days of treatment with either product [74].

In summary, although just a few studies have been reported concerning the use of CNBs for the treatment of AE, the results are promising and it is possible that in the future these compounds can be used to substitute current treatments with undesirable side effects (e.g., the use of corticosteroids).

#### 4.3.4. Atopic Dermatitis

Atopic dermatitis (AD) is a chronic skin disease characterized by impaired epidermal barrier function combined with a chronic Th2-type inflammatory state [75]. This relapsing/remitting inflammatory disease is characterized by weepy red plaques in the acute stage, and lichenified thick plaques in the chronic stage, that cause intense pruritus and discomfort. Even though its pathogenesis is not well understood yet, it is believed to be a result of various factors, such as immune dysregulation, epidermal and sebaceous barrier disruption, altered sensation to itch stimuli, and impaired microbial defense [30].

Treatment for AD mainly focuses on anti-inflammatories, such as topical steroids and calcineurin-inhibitors, barrier repair using moisturizers, and the reduction in microbial colonization. Nonetheless, multiple studies suggested the targeting of the skin ECS, particularly CB1R, as a possible treatment for AD [49]. Studies in mice showed that the activation of CB1R in skin cells improved the epidermal barrier function, decreased a Th2-type inflammatory response and suppressed mast cells [75,76,77]. Thus, topical preparations containing ECB receptor agonists or degradation inhibitors may have a high therapeutic value in AD [30] (see Table 3).

Several clinical trials have also provided evidence on the effectiveness of topical CNBs to treat AD. For example, Del Rosso [79] reported the results of a randomized trial involving 43 patients, adults and children, on the efficacy of a PAE-containing non-steroidal cream to treat AD. The results showed that the treatment with a combination of the PAE-based cream with a mid-potency topical corticosteroid (0.1% clocortolone pivalate) led to faster skin clearance than the control, treated with a corticosteroid cream only. Additionally, the use of the PAE cream increased the time between flares by approximately 28 days compared to the control. Eberlein et al. [80] conducted a multinational, multicentre, observational, non-controlled, prospective cohort study, in which patients with ages ranging from 2 to 70 years were treated with a PEA-based cream. Data from 2456 patients were analyzed and showed that substantial relief of objective and subjective symptoms of AD were achieved after regular skincare with the studied formulation. The recorded decline in pruritus and loss of sleep indicated a gain in the quality of life in these patients, and the reduced need for topical corticosteroids was also an important finding, due to the side effects of these drugs. Adverse events proven to be ‘definitely’ or ‘probably’ related to the cream use occurred in only 2.3% of the subjects and included pruritus, burning, and erythema. No serious side effects were reported. An earlier study by Callaway et al. [78] compared the use of dietary hemp seed oil and olive oil in a 20 week randomized, single-blind crossover study with AD patients. In this study, the treatment was oral and it was reported that a daily ingestion of 30 mL hempseed oil caused significant changes in plasma fatty acid profiles, and decreased skin dryness, irritation and itchiness, unlike olive oil. The authors hypothesized that this effect was due to the high amount of PUFAs present in the hempseed oil. Furthermore, no patients experienced any adverse reaction to either oil during the treatment period. Recently, a polycaprolactone (PCL) patch was developed with the aim to study the long-term release of hemp seed oil on a skin model and on the skin of three human volunteers [81]. The results showed up to 55% of oil release within 6 h, while the moisturization of the volunteers’ skin increased around 25%. This controlled oil release is crucial to maintain skin moisturization over time, thus the patches were proposed as novel, easy-to-use therapeutic devices for the treatment of AD.

In conclusion, several studies and clinical trials have shown that CNBs and CNB-containing oils have been helpful in alleviating AD symptoms such as pruritus, irritation and skin dryness.

#### 4.3.5. Psoriasis

Psoriasis is an autoimmune inflammatory hyperproliferative skin disease, notable for the manifestation of lesions (‘scales’) that develop within the epidermis, originated by an extremely fast turnover of epidermal keratinocyte proliferation, accompanied by the infiltration and increased expression of proinflammatory mediators into the skin. The pathogenesis of psoriasis seems to combine genetics and environmental factors and develops due to pathological interactions between immune skin cells and epidermal keratinocytes, resulting in increased inflammation (due to production of cytokines such as IL-17, IL-22 and TNF-α) and excessive proliferation of keratinocytes, and leading to the characteristic skin alteration called psoriatic plaque [37,95,96]. It affects between 2% and 3% of the world population and presents significant morbidity, often causing anxiety and depression in patients [97]. The cutaneous ECS inhibits cell growth and angiogenesis, leading to skin cell apoptosis [50], thus it is not unexpected that CNBs have shown promising results in helping to treat psoriasis, a hyperproliferative inflammatory skin disease.

The inhibition of keratinocyte proliferation by several CNBs (THC, CBD, CBN and CBG) was reported by Wilkinson et al. [84] in an in vitro study using a hyper-proliferating human keratinocyte cell line. The results showed proliferation inhibition in a concentration-dependent manner, independent of CBR activation, with the authors suggesting a mechanism involving the PPAR receptor. A later study, by Ramot et al. [85], reported a different inhibitory mechanism, occurring through downregulation of keratins K6 and K16 expression by CB1R activation. The in situ studies used organ-cultured human skin, and showed that stimulation with a CB1R specific agonist decreased expression of the keratins, which are upregulated in psoriatic skin. A similar result was obtained in in vitro studies using human HaCaT keratinocytes, with the CB1R agonist decreasing the expression of K6 at the transcription and translation levels.

Cannabinoids may also be promising in psoriasis therapeutics due to their anti-inflammatory effects. Namazi [98] reported that CNBs inhibited antigen processing in macrophages, macrophage/T-cell interaction, and release of pro-inflammatory cytokines (IL-2 and TNF-α) and nitric oxide from immune cells. Since psoriasis is characterized by a type 1 cytokine pattern (where IFN-γ, IL-2, IL-1 and TNF-α are predominantly expressed), which occurs following the presentation of the antigen to CD4+ T lymphocytes and resulting in stimulation of keratinocyte proliferation and expression of adhesion molecules, the authors hypothesized that CNBs could have therapeutic efficacy against psoriasis, given their inhibitory effect of the inflammatory mechanisms. Derakhshan and Kazemi [99] also suggested a possible therapeutic action of CNBs in psoriasis due to their keratinocyte antiproliferative action and the anti-inflammatory role due to vagal nerve stimulation followed by acetylcholine release and immunomodulation via inhibition of TNF-α production by cytokine-producing macrophages [15,99].

Still related to the antiproliferative and anti-inflammatory actions of CNBs, it was reported that the gene NRIP1, which was previously shown to be an important target gene of CBD (with lipogenic effect in acne and seborrhea disorders), was overexpressed in psoriatic skin, and that its downregulation in HaCaT keratinocytes significantly suppressed their proliferation. Furthermore, the inhibition of NRIP1 also reduced the expression of p65 NF-κB and the release of IL-17, thus suggesting that NRIP1 may be a multifaceted therapeutic target in psoriasis [100]. Additionally, in the previously discussed study of the pathology of acne and seborrhea [64], it was reported that, in cultured human sebocytes, CBD negatively regulated NRIP1 in a TRPV4-dependent pathway. Therefore, it can be hypothesized that CBD exerts its anti-inflammatory effects on psoriasis via the activation of the same signaling pathway [41].

Norooznezhad and Norooznezhad [86] suggested targeting angiogenesis, another process involved in psoriasis pathogenesis, with the synthetic cannabinoid JWH-133. This molecule has antiangiogenic and anti-inflammatory properties, inhibiting the production of several angiogenic growth factors (e.g., HIF-1 α, VEGF, MMPs, and bFGF) and cytokines (e.g., IL-8 and IL-17), thus it can target two main features of psoriasis pathogenesis, inflammation and angiogenesis.

A few clinical studies have been reported for the treatment of psoriasis with CNBs. In 2019, a patent was launched for the treatment of psoriasis with the application of different topical formulations (ointment, gel, liquid, spray, and powder) containing CNBs, mainly CBD and CBG (natural or synthetic), in concentrations of 3–20%. The application of the formulation in the affected areas led to a dose-dependent improvement in psoriasis, while controls that received placebo oil showed no improvement. The authors suggested a possible T cell (Th1 and Th2) rebalancing mechanism, as well as a direct CBG inhibition of keratinocyte proliferation [87]. Friedman et al. [88] reported a case study of a 33-year-old male with psoriasis, which started in the face and then spread to several body areas. The patient was treated with cream, soap, and hair oil, all containing THC (5 mg/mL) distillate and reported improvement within 2 days after initiating treatment. Seven months after, the patient reported only using the products for maintenance, and that any flare of the disease could be quickly controlled with the treatment.

In summary, these findings do support a potential role for CNBs in the treatment of psoriasis, possibly involving a combination of their antiproliferative, anti-inflammatory and antiangiogenesis properties. This possibility is highly relevant, since antipsoriatic medications are often associated with adverse side effects [101], and so, an ongoing search for safer agents that can be used alone or in combination with current antipsoriatic drugs is imperative.

#### 4.3.6. Pruritus

Pruritus, also known as itch, is an unpleasant localized or generalized, very common symptom in inflammatory skin diseases, contributing significantly to the impaired quality of life of affected patients, since it is considered one of the most bothersome symptoms [25,50]. Despite the existence of multiple possible antipruritic regimens, they often show low efficacy rates, and thus new treatment options that may improve the lives of those affected are always welcomed.

The ECS plays an essential part in the central and peripheral processing, and skin-derived sensory manifestations, such as pain and pruritus. Cannabinoid receptors have been identified in sensory nerve terminals and/or inflammatory cells, thus cannabinoid agonists and/or ECBs seem a rational therapeutic option for pruritus, especially in patients who failed to improve with other treatment modalities [50,57]. In fact, these compounds have shown powerful analgesic and antipruritic effects in humans and animals, through the activation of CB1R and/or CB2R, and possibly other receptors (e.g., TRPV1) [50]. As an example, the previously described study by Eberlein et al. [80] reported how the use of a cream containing PEA significantly decreased objective and subjective symptoms of ACD, including pruritus. Additionally, Schlosburg et al. [102] observed that the suppression of the neuronal FAAH reduced the scratching response through the inhibition of AEA degradation and activation of CB1R.

Several clinical studies have also shown a reduction in pruritus caused by dermatologic (AD, psoriasis, asteatotic eczema, and ACD) and systemic (uremic pruritus and cholestatic pruritus) diseases [103] (Table 3). A preliminary study on the effect of an AEA/PEA cream with structured physiological lipids was reported by Szepietowski and co-workers [56]. The trial was completed by 21 uremic pruritus patients and, after 3 weeks of treatment, global pruritus and xerosis were evaluated. The results showed a good tolerance to the product and a complete elimination of pruritus in 38% of patients, while xerosis scores were significantly reduced in 81% of the patients. The product was well tolerated by all patients and no side effects were observed. Another low-scale clinical trial enrolling 22 patients studied the effect of an emollient cream containing only PEA and showed antipruritic action in 64% of the cases and an average reduction in itch of 86%. The cream was well tolerated by all patients [57]. A short communication by Visse et al. [83] also reported the effects of a PEA lotion in subjects with chronic pruritus (100 participants). The study compared the efficiency of a derma-membrane system (DMS)-based lotion containing PEA with the vehicle, in terms of symptom improvement, cosmetic acceptance and quality of life. In contrast to previous studies, however, the results did not show significant differences between the two lotions concerning pruritus, even if there was a slight improvement. The lotion containing PEA significantly decreased the stinging sensation compared to the control. The cosmetic properties of both lotions were considered good, and their regular application improved dry skin. Adverse effects related to worsening of skin symptoms were reported by 13.3% in each group (PEA-treated and control). The authors suggested performing further trials to evaluate a possible placebo effect (by including an untreated group control, for example) and using the reduction in stinging as the main criterion outcome. Dvorak et al. [82] studied the effects of a CB1R/CB2R synthetic agonist, HU210, in a histamine-induced itch model. The agonist was peripherally administered to the subjects either by dermal microdialysis (5 mM) or by skin patch (50 mM) and skin blood flow, widespread flare reaction, extravasation of plasma proteins and perceived itch were monitored. The results showed that HU210 significantly reduced histamine-induced itch and that this effect was not due to antihistaminergic activity, leading the authors to suggest the possible use of this and similar compounds in the treatment of sensitive, inflamed and/or itchy skin when other treatments are not efficient.

Because dry skin can be the leading cause or a promoter for pruritus or skin diseases such as dermatitis, the application of formulations containing CNBs that stimulate CB2R (CB2R agonists) in the SGs, and/or agents that increase the local production of ECBs and/or inhibit their degradation (e.g., FAAH and/or MAGL inhibitors) in the SGs [104], can increase fat production in the SGs, thus relieving dry skin and pruritus [50]. However, it is important that these topical medications are made from ECS-acting substances that, on absorption to the blood, do not penetrate the brain and that henceforth will not cause psychoactive effects. The use of FAAH/MAGL inhibitors has been referred to in some studies but these were administered systemically [102,105], thus they will not be discussed here.

In summary, the modulation of the skin ECS by targeting CNB receptors and/or metabolic enzymes seems a promising approach to decrease pruritus, a symptom associated with several of the skin diseases discussed in the previous sections.

## 5. Legislation on Cannabis Use and Available Therapeutics

Phytocannabinoids have physiological and often psychoactive activity, the most potent being THC, as discussed. Due to these effects, several preparations of *C. sativa* are consumed as drugs, and cannabis is considered the most popular illicit drug of the twenty-first century [106]. For this reason, the use of cannabis and its derivatives must be regulated internationally but the rules vary.

Cannabis plants and products are obligatorily controlled by international laws, with some permissions for medical and industrial use [107]. The use of cannabis for medical purposes is controlled internationally in accordance with three international conventions of the United Nations (UN): the 1961 Single Convention on Narcotic Drugs (amended in the 1972 Protocol), the 1971 Convention on Psychotropic Substances, and the 1988 Convention Against Illicit Traffic in Narcotic Drugs and Psychotropic Substances [108]. These treaties impose certain requirements on signatory countries in order to consent to the use of medicinal cannabis and its derivatives under international control, requiring stricter regulation of these cannabis-based medicines [109]. However, the low risk of CBD dependence and abuse, the fact that this CNB has potential benefits in certain pathologies and is not explicitly listed in the UN convention tables, led to an ambiguous and heterogeneous European regulatory environment for CBD [110]. Thus, there are numerous CBD products with claims for medicinal purposes (describing health benefits, but often without evidence), such as capsule supplements for various diseases and cosmetics (for example, hemp oils), which are manufactured and distributed without regulatory supervision and sometimes with unverified content [111,112].

In North America, some of the USA states legalized the use of cannabis for medical purposes (chronic pain, multiple sclerosis, terminal cancer) in the mid-1990s. As of May 18, 2021, 36 states and four territories have legalized cannabis products for medical use; but at the federal level, cannabis is still classified as a Schedule I substance (having high dependency potential and no accepted medical use) [113], thus its use is prohibited for all purposes. Canada presented a medical cannabis program in 1999, which has evolved since then. Since the early 2000s, several other countries approved cannabis for medical use, albeit with restrictions, including several European countries [14].

In Europe, there are no continent-wide regulations but there are three routes to obtain a medicine use authorization: (i) centralized procedure, where the European Medicines Agency (EMA) approves the medicine marketing; (ii) decentralized procedure in individual countries, obtained from the countries’ health agencies; and (iii) mutual recognition, where an European Union (EU) country authorizes the marketing of a product which is already authorized in another EU country.

Currently, there are only a few cannabis-based medicines in the market (summarized in Table 4) and none of these aim at the treatment of dermatological diseases. The only cannabis-based medicine which was granted marketing authorization by the EMA for use in the EU is Epidyolex [114]. This cannabis-based drug contains a purified form of CBD, and is indicated for the treatment of seizures associated with Lennox–Gastaut syndrome and Dravet syndrome in patients 1 year of age and older [115,116]. However, Sativex, Canemes, and Marinol/Syndros are sold in several EU countries as approved medicines, although not centrally approved by the EMA [114,117,118]. Additionally, Bedrocan, an EU-Good Manufacturing Practice (GMP) company based in The Netherlands, supplies high-quality, pharmaceutical-grade cannabis, which can be used as raw material and an active pharmaceuticals ingredient [119]. However, most countries still prohibit the use of herbal cannabis, with only Canada, Israel, Germany and The Netherlands fully authorizing its use for medical purposes [117].

Even if there is still limited evidence on the effectiveness and safety of cannabis for the treatment of dermatologic diseases, dispensaries have been making claims that are, in most cases, unsubstantiated, and that can be found online, fueling the interest of patients. A recent survey of dispensary websites in the USA, Canada and Europe showed that these suggest the use of topical cannabis to treat pain, inflammation, dryness, and eczema, for example [122]. Additionally, several of the topical preparations that are sold over the counter can cause immunologic contact urticaria and ACD due to the presence of allergens, including other botanicals [123]. The legal framework for the use of *Cannabis sativa* products, including topical formulations for cosmetics and medical use, must still evolve until being firmly established.

## 6. Conclusions and Future Perspectives

Cannabis and cannabinoid-based products seem to have promising applications in skincare, both in the cosmetics industry and for the topical treatment of skin diseases such as pruritus, inflammatory diseases, and even skin cancers. The studies reviewed here suggest that CNBs and CNB receptor modulators (e.g., agonists/antagonists), can have a therapeutic action in several inflammatory skin diseases, due to their antiproliferative, immunomodulatory and anti-inflammatory actions. However, to further explore such possibilities, our knowledge of the cutaneous cannabinoid system must expand. Because CNBs can bind to multiple receptors (not necessarily limited to CBRs), with varying affinities, or possibly even acting in a receptor-independent manner, they can lead to biological outcomes that currently cannot be reliably predicted, challenging the approval of cannabis-based therapies.

Although approved CNB-based medicines to treat skin disorders are not yet available in the market, several studies have provided preliminary evidence of the potential benefits of these compounds in these conditions, as reviewed in this paper. However, most research on the use of topical cannabis has been performed in vitro or in vivo using animal models. The few available clinical trials are usually small, and lacking rigorous design (for example, greatly varying in formulation, route of administration, dosage, and frequency of use), not providing enough data concerning safety and efficacy. Thus, there is a clear need for high-quality randomized controlled trials that, as per the requirement of the International Council for Harmonisation of Technical Requirements for Pharmaceuticals for Human Use (ICH) Good Clinical Practice (GCP) E6, to provide safety data supporting the study design elements (e.g., participant population, dosing, and expected adverse effects), and an outline to a post-study safety monitoring approach, in order to completely assess the efficacy and safety of these compounds, before their use can be authorized for the treatment of dermatological diseases [115]. Even though the undertaking of human studies with *C. sativa* L.-derived pharmaceuticals is necessary to demonstrate their efficacy and safety in various clinical settings, those already performed highlighted some unique challenges, causing some apprehension, particularly with ethics and governance committees, when it comes to the endorsement of new trials using cannabis-based drugs, and consequently rising barriers that are slowing down the progress in their use in medicine [124].

A search on PubMed clearly shows an increasing trend in the literature of studies on cannabis/CNBs and skin diseases. Thus, it is likely that, as knowledge increases, there will be developments in the legal status of cannabis-based medicines, with more countries approving their use. It is important to separate the use of cannabis for recreational use from its medical use. In fact, the recreational use of this drug can lead to several disorders and is a public health concern [125].

## Figures and Tables

**Table 1:** The different classes of cannabinoids, and some examples [15].

Endocannabinoids	Phytocannabinoids	Synthetic Cannabinoids
2-Arachidonoylglycerol (2-AG) Anandamide (AEA) N-Palmitoylethanolamide (PEA) Oleoylethanolamide (OEA)	Cannabidiol (CBD) Cannabigerol (CBG) Cannabichromene (CBC) Cannabinodiol Cannabitriol ** Cannabielsoin Cannabicyclol Cannabinol (CNB-ol) Δ9-Tetrahydrocannabinol (THC) * Δ-9-Tetrahydrocannabivarin (THCV) Δ-9-Tetrahydrocannabinolic acid (THCA) (-)-Δ8-trans-tetrahydrocannabinol *	JWH-133 (R)-Methanandamide HU-308 JTE-907 SR 144,528

* psychoactive substance; ** unknown psychoactivity status.

**Table 2:** The different classes of cannabinoids located in the skin, and some examples [15,37,47,48].

	Type of Receptor	Name	Location in Skin	Main Ligands (ECBs and pCNBs)	Interaction
Main receptors	G-protein-coupled receptor	CB1R	Sensory nerves, hair follicles, immunocytes, keratinocytes, melanocytes, sebaceous glands	AEA	Weak partial agonist
				CBD	Negative allosteric modulator
				THC	Partial agonist
				THCV	Antagonist
		CB2R	Immunocytes, keratinocytes, melanocytes, sensory neurons, sebaceous glands	AEA	Weak partial agonist
				CBD	Inverse agonist
				THC, THCV	Partial agonist
Secondary receptors	Transient potential ion channels	TRPV-1	Sweat and sebaceous glands, keratinocytes, melanocytes, nerves, immunocytes	AEA, THC	Weak agonist
				CBD, CBGA, CBGV, THCV	Strong agonists
				CBG	Agonist
		TRPV-2	Sensory nerves, keratinocytes, immunocytes, fibroblasts	CBD, CBG, CBGV, THC, THCA, THCV	Strong agonists
		TRPV-3	Hair follicle, immunocytes, keratinocytes, fibroblasts, sensory nerves	CBD	Agonist (action similar to the typical agonist carvacrol)
				THCV	Strong agonist
				THC	Weak agonist
		TRPV-4	Immunocytes, keratinocytes, fibroblasts, sensory nerves	AEA, 2-AG	Agonists (indirect activation)
				CBDV, THCV	Strong agonists
				THC	Weak agonist
		TRPA1	Immunocytes, keratinocytes, fibroblasts, sensory nerves	AEA, THC	Agonist
				CBD, CBC, CBN	Strong agonists
		TRPM8	Immunocytes, keratinocytes, fibroblasts, sensory nerves	AEA, THC, THCA, CBD, CBN	Strong antagonists
	Peroxisome proliferator-activated receptors	PPAR-α	Immunocytes, keratinocytes, melanocytes	THC, CBGA	Agonists
				CBDA, CBG	Partial agonists
		PPAR-γ	Keratinocytes, melanocytes, fibroblasts, hair follicles	THC, CBD	Agonists
	Serotonin receptors	5-HT1A	Immunocytes, keratinocytes, melanocytes, fibroblasts	CBG	Strong antagonist
				CBD, THCV, CBDA	Agonists
		5-HT2A	Immunocytes, keratinocytes, melanocytes, fibroblasts, sensory nerves	CBD	Partial agonist
		5-HT3	Immunocytes, keratinocytes	CBD, THC	Antagonists

AEA, anandamide; 2-AG, 2-arachidoynyl-glycerol; CB1R/CB2R, main cannabinoid receptors; CBC, cannabichromene; CBD, cannabidiol; CBDA, cannabidiolic acid; CBG, cannabigerol; CBGA, cannabigerolic acid; CBGV, cannabigerovarin; CBN, cannabinol; ECB, endocannabinoid; pCB, phytocannabinoid; 5-HT, serotonin receptors; PPAR, peroxisome proliferator-activated receptors; THC, trans-Δ-9-tetrahydrocannabinol; THCA, Δ9-tetrahydrocannabinolic acid; THCV, tetrahydrocannabivarin; TRPV, transient potential ion channels receptors. Weak agonist means a substance which, upon binding to a receptor, is only able to elicit a low response; strong agonist is the opposite. A partial agonist is only able to induce sub-maximal activation of a receptor, independently of its concentration. An indirect agonist is a compound that can induce a certain response not by directly binding to a receptor but through an indirect mechanism.

**Table 3:** Summary of research and clinical studies on the use of cannabinoids to treat dermatological disorders.

Disease	Type of Study	Short Description	Results	Ref.
Acne and seborrhea	In vitro lab research	Production and effects of ECBs in cultured human SZ95 sebocytes.	Cells produced AEA and 2-AG and expressed CB2R but not CB1R. Lipid synthesis and apoptosis-driven cell death via CB2R were upregulated by AEA and 2-AG.	[63]
	In vitro lab research	Effect of CBD in cultured human SZ95 sebocytes and human skin organ culture.	CBD inhibited the lipogenic actions of several compounds, suppressed sebocyte proliferation and had anti-inflammatory action, inhibiting the NF-κB signaling pathway.	[64]
	Single-blind comparative study (11 participants)	Effect of *C. sativa* seed extract cream (3%) on acne symptoms.	Decreased sebum and erythema levels.	[65]
	In vitro lab research	Effect of cannabinoids in cultured human SZ95 sebocytes.	CBC, CBDV suppressed AA-induced seborrhea lipogenesis. THCV inhibited sebocyte proliferation and AA-induced seborrhea lipogenesis. CBG, CBGV had pro-lipogenic and pro-acne actions.	[66]
	In vitro lab research	Effect of hemp seed extracts on human HaCaT keratinocytes and primary human sebocytes.	Hemp seed hexane extracts (HSHE) had antimicrobial activity against C. acnes, anti-inflammatory, anti-lipogenic, and collagen-promoting properties.	[67]
	Clinical trial (368 participants)	Effect of BTX 1503 (topical solution with 5% CBD).	After 12 weeks of treatment there was a 40% reduction in acne lesions.	[68]
Allergic contact dermatitis (ACD)	In vivo lab research	Effect of CB2R antagonists/reverse agonists in a mice ear ACD model.	Mice ears showed swelling within 1 day after being treated with a 2-AG analogue and within 1-8 days after treatment with a CB2R agonist. Oral administration of a CB2R antagonist or reverse agonist decreased the swelling in these ACD models and also in an DNFB-induced ACD model.	[69]
	In vivo lab research	Effect of CB1R/CB2R antagonists on oxazolone-induced ACD in mice ears.	Oxazolone-challenged mice ears had increased concentrations of 2-AG. Treatment with a CB2R antagonist (but not CB1R antagonist) suppressed the inflammatory response.	[70]
	In vivo lab research	Response of WT and CB1R/CB2R knockout mutant mice to DNFB-induced ACD.	Mice knocked-out for CB1R/CB2R showed exacerbated allergic inflammation to DNFB-induced ACD. Antagonists of CBRs led to exacerbated allergic inflammation in WT mice, while agonists attenuated the inflammatory response. Mice deficient in FAAH had increased concentrations of AEA and reduced allergic responses.	[44]
	In vitro and in vivo lab research	Production and effect of PEA in an DNFB-induced ACD mice model and HaCaT keratinocytes.	Endogenous production and exogenous application of PEA decreased symptoms of DNFB-induced ACD. Keratinocytes induced with poly-(I:C) had higher levels of PEA, and exogenous PEA treatment inhibited the secretion of pro-inflammatory mediators, an effect reversed by TRPV1 antagonists, but not PPAR-α or CB2R antagonists.	[71]
	In vitro and in vivo lab research	Effect of THC in a DNFB-induced mice model of ACD	Topical application of THC decreased ear swelling independently of CB1R/CB2R by decreasing the secretion of IFN-γ by T cells and myeloid immune cell infiltration. In vitro, THC inhibited the IFN-γ-dependent production of chemokines by mice primary epidermal keratinocytes.	[72]
	In vitro lab research	Effect of CBD in poly-(I:C)-stimulated human HaCaT keratinocytes.	Treatment with CBD increased AEA levels and inhibited the production of MCP-2, IL-6, IL-8 and TNF-α. This was reversed by treatment with CB2R and TRPV1 antagonists.	[73]
Asteatotic eczema	Randomized double-blind controlled study (60 participants)	Compare PEA/AEA (0.3%/0.21%) emollient cream with a traditional emollient.	Improved scaling, dryness, and itching at day 28. Increased skin hydration (measured by change in capacitance of the skin surface), back to normal levels in 7 days. No difference in TEWL between PEA/AEA and control creams.	[74]
Atopic dermatitis (AD)	In vivo lab research	Research the role of CB1R in fluorescein isothiocyanate (FTIC)-induced AD in mice ears.	Mice knocked out for CB1R globally or in keratinocytes had enhanced responses to FTIC and delayed epidermal barrier repair. Inflamed ear tissue had higher pro-inflammatory cytokines and chemokines mRNA level, and higher eosinophil activity. CB1R-deficient epidermal keratinocytes secreted higher levels of TSLP and CCL8, inducing a Th2-type skin inflammation.	[75]
	In vivo lab research	Effects of CB1R agonists on skin inflammation in acute and chronic oxazolone-induced AD animal models.	The topical application of the agonists accelerated the recovery of the epidermal barrier function and had anti-inflammatory effects, confirmed by histological studies.	[76]
	In vivo lab research	Effects of CB1R agonists (AEA derived) on mast cell activation.	CB1R agonists suppressed mast cell proliferation in a dose-dependent manner, suggesting an important role for CB1R plays in the modulation of antigen-dependent IgE-mediated mast cell activation.	[77]
	Single-blind crossover (20 participants)	Effect of dietary hempseed oil.	Improvement of skin dryness and itchiness. Decrease in dermal medication usage.	[78]
	Investigator-blinded comparative study (43 participants)	Effect of PEA-containing non-steroidal cream.	Increased the mean time to the next flare by an average of 28 days, compared to moisturizer cream (both combined with a topical corticosteroid cream).	[79]
	Cohort (2546 participants)	Effect of emollient cream containing PEA.	Decreased severity, flare-ups and use of topical steroids. Improved symptoms, disease tolerance and sleep.	[80]
	In vitro (skin model); in vivo (3 human volunteers)	Effect of PCL patch with hemp seed oil.	Long-term release of hemp seed oil from the patches (55% over 6 h) and 20–25% increase in skin hydration.	[81]
Chronic pruritus	Double-blinded comparative study (12+6 participants)	Effect of cannabinoid receptor agonist HU210 (skin patch or microdialysis).	Reduced experimentally-induced itch and attenuated increase in blood flow.	[82]
	Clinical trial (21 participants)	Effect of AEA/PEA cream with Derma Membrane Structure (DMS) in uremic pruritus.	After a 3 week therapy, there was a complete elimination of pruritis in 38% patients and reduction in xerosis in 81% patients. The product was well tolerated by all patients.	[56]
	Cohort (22 participants)	Effect of emollient cream containing PEA.	Reduced subjective severity of itch (average reduction of 86%). Antipruritic effect observed in 64% of the cases.	[57]
	Single-blind comparative study (100 participants)	DMS-based dermatocosmetic lotion containing PEA.	No significant differences between DMS-based PEA lotion group and control group concerning itch, quality of life, or cosmetic acceptance.	[83]
Psoriasis	In vitro lab research	Effect of THC, CBD, CBN, CBG on keratinocyte proliferation.	Inhibition of cell proliferation, concentration-dependent and independent of CB1R/CB2R.	[84]
	In vitro and in situ lab research	Effect of CB1R agonist in the levels of keratins K6 and K16.	Downregulation of keratins expression in situ (organ-cultured human skin) and in vitro (HaCaT keratinocytes), suggesting the involvement of CB1R in the process.	[85]
	Hypothesis	Use of JWH-133 (synthetic cannabinoid) as a therapy for psoriasis.	Study of JWH-133, a potent antiangiogenic and anti-inflammatory agent, for the treatment of psoriasis.	[86]
	Patent	Effects of CBD/CBG oil in 2 psoriatic patients.	16–33% reduction in lesions observed after 6 weeks.	[87]
	Case study	Effect of products with THC distillate in a 33-year-old psoriasis patient.	Treatment with cream, soap and oil improved psoriasis symptoms as early as 2 days after beginning. Flare-ups could be controlled by reinitiating the treatment.	[88]

AA, arachidonic acid; ACD, allergic contact dermatitis; AD, atopic dermatitis; AEA, anandamide; 2-AG, 2-arachidoynyl-glycerol; CBC, cannabichromene; CBD, cannabidiol; CBDV, cannabidivarin; CBG, cannabigerol; CBGV, cannabigerovarin; CBN, cannabinol; CB1R/CB2R, G protein-coupled CNB (main) receptors; DMS, Derma Membrane Structure; DNFB, 2,4-dinitrofluorbenzene; ECB, endocannabinoid; FAAH, fatty acid amide hydrolase; FTIC, fluorescein isothiocyanate; HSHE, hemp seed hexane extracts; IFN-γ, interferon γ; IL, interleukin; MCP-2, monocyte chemotactic protein-2; PCL, polycaprolactone; PEA, N-palmitoylethanolamine; PPAR-α, peroxisome proliferator-activated receptor α; TEWL, transepidermal water loss; THC, *trans*-Δ-9-tetrahydrocannabinol; THCV, tetrahydrocannabivarin; TNF-α, tumor necrosis factor α; TRPV1, transient potential channel receptor 1; TSLP, thymic stromal lymphopoietin; WT, wild type.

**Table 4:** Authorized cannabis-based medicines [107,117,118,120,121].

Brand Name	Active Ingredients	Description	Indications	Dosage Forms	Countries Approved
Sativex^®^	Nabiximols	Plant based: THC/CBD (~1:1)	Spasticity due to multiple sclerosis	Oromucosal spray	UK, Norway, some EU countries, Canada
Marinol^^®^^, Syndros^®^	Dronabinol *	Synthetic THC	Treatment of nausea and vomiting due to chemotherapy, anorexia due to AIDS	Gelatine capsules (Marinol), oral solution (Syndros)	USA, EU countries, Canada, others
Cesamet^®^ Canemes^®^	Nabilone **	Synthetic cannabinoid similar to THC	Treat nausea and vomiting due to chemotherapy in cancer patients; chronic pain management	Capsules	USA, Canada, some EU countries
Epidyolex^®^ (EU) Epidiolex^®^ (USA)	CBD	Purified CBD	Seizures associated with Lennox–Gastaut syndrome, Dravet syndrome	Oral solution	EU, USA
Bedrocan [119]	Several	Plant material; (5 plant varieties available)	Various	Dried flower tips (sometimes powdered)	Australia, South Africa, some European countries

* The WHO name (International Non-proprietary Name, INN) for a specific variant of Δ9 -THC that occurs naturally in the *Cannabis* plant is dronabinol, and the terms are used interchangeably in the literature. Chemically synthesized dronabinol is marketed as Marinol. ** (Cesamet) is a synthetic cannabinoid not occurring in nature.

## Data Availability

Not applicable.
