# Correction: Martins et al. Cannabis-Based Products for the Treatment of Skin Inflammatory Diseases: A Timely Review. *Pharmaceuticals* 2022, *15*, 210

**DOI:** 10.3390/ph15070849

**Published:** 2022-07-11

**Authors:** Ana M. Martins, Ana L. Gomes, Inês Vilas Boas, Joana Marto, Helena M. Ribeiro

**Affiliations:** 1 Research Institute for Medicines (iMed.ULisboa), Universidade de Lisboa, 1649-003 Lisbon, Portugal; hribeiro@campus.ul.pt; 2 Faculdade de Farmácia, Universidade de Lisboa, 1649-003 Lisbon, Portugal; analucia199867@gmail.com (A.L.G.); inesvb97@gmail.com (I.V.B.)

The authors would like to make the following corrections about the published paper [1]. The changes are as follows:

**Missing Citation**

In the original publication, refs. [2,3] was not cited. The citation has now been inserted in **4. The Skin Endocannabinoid System and the Use of Cannabinoids as a Potential Treatment for Skin Inflammatory Diseases**, *4.1. The Skin Endocannabinoid System*, **Table 2** caption. Newly add references as [47,48] in the original paper, the order of some reference citation has been adjusted correspondingly.

“Table 2. The different classes of cannabinoids located in the skin, and some examples [15,37,47,48].”

The authors apologize for any inconvenience caused and state that the scientific conclusions are unaffected. This correction was approved by the Academic Editor. The original publication has also been updated.
